# Sarcoidosis and Tuberculosis Cytokine Profiles: Indistinguishable in Bronchoalveolar Lavage but Different in Blood

**DOI:** 10.1371/journal.pone.0038083

**Published:** 2012-07-16

**Authors:** Muhunthan Thillai, Christian Eberhardt, Alex M. Lewin, Lee Potiphar, Suzie Hingley-Wilson, Saranya Sridhar, Jonathan Macintyre, Onn Min Kon, Melissa Wickremasinghe, Athol Wells, Mark E. Weeks, Donald Mitchell, Ajit Lalvani

**Affiliations:** 1 Department of Respiratory Medicine, National Heart and Lung Institute, Imperial College London, London, United Kingdom; 2 Biostatistics Group, Department of Epidemiology and Public Health, Imperial College London, London, United Kingdom; 3 Department of Respiratory Medicine, St. Mary’s Hospital, London, United Kingdom; 4 Interstitial Lung Unit, Royal Brompton Hospital, Imperial College London NHS Healthcare Trust, London, United Kingdom; 5 Molecular Haematology and Cancer Biology Unit, UCL Institute of Child Health, London, United Kingdom; University of Cape Town, South Africa

## Abstract

**Background:**

The clinical, radiological and pathological similarities between sarcoidosis and tuberculosis can make disease differentiation challenging. A complicating factor is that some cases of sarcoidosis may be initiated by mycobacteria. We hypothesised that immunological profiling might provide insight into a possible relationship between the diseases or allow us to distinguish between them.

**Methods:**

We analysed bronchoalveolar lavage (BAL) fluid in sarcoidosis (n = 18), tuberculosis (n = 12) and healthy volunteers (n = 16). We further investigated serum samples in the same groups; sarcoidosis (n = 40), tuberculosis (n = 15) and healthy volunteers (n = 40). A cross-sectional analysis of multiple cytokine profiles was performed and data used to discriminate between samples.

**Results:**

We found that BAL profiles were indistinguishable between both diseases and significantly different from healthy volunteers. In sera, tuberculosis patients had significantly lower levels of the Th2 cytokine interleukin-4 (IL-4) than those with sarcoidosis (p = 0.004). Additional serum differences allowed us to create a linear regression model for disease differentiation (within-sample accuracy 91%, cross-validation accuracy 73%).

**Conclusions:**

These data warrant replication in independent cohorts to further develop and validate a serum cytokine signature that may be able to distinguish sarcoidosis from tuberculosis. Systemic Th2 cytokine differences between sarcoidosis and tuberculosis may also underly different disease outcomes to similar respiratory stimuli.

## Introduction

There are numerous anecdotal case reports of patients with shared respiratory exposures who subsequently develop different diseases. One such intriguing study involves monozygotic twins. One twin presented with dyspneoa, a pleural effusion, strongly positive Mantoux and negative Kveim test, and subsequently responded appropriately to anti-tuberculous therapy. The other twin denied respiratory symptoms but a chest radiograph revealed bilateral hilar lymphadenopathy, a Kveim test was positive and the clinical course was in keeping with sarcoidosis [1].

Mycobacterial involvement in sarcoidosis pathogenesis remains a contentious issue. Supporting evidence includes similarities in clinical and radiographic picture [2], identification of mycobacterial DNA in sarcoid granulomas [3] and the finding in some sarcoidosis cohorts of peripheral blood and bronchoalveolar lavage (BAL) INFγ-mediated T-cell responses towards a mycobacterial proteins such as KatG [4]. Conversely, evidence against a relationship includes the marked differences in extra-pulmonary disease sites and treatment strategy [2], and the absence of INFγ-mediated T-cell responses towards the immunodominant mycobacterial proteins ESAT-6 and CFP-10 in large worldwide sarcoidosis studies [5]–[7].

The T-cell profile in sarcoidosis BAL cells is biased towards Th1 cytokines [8] with similar findings in tuberculosis [9]. This picture is distinct from that seen in atopic asthma which exhibits a Th2 bias [10] or hypersensitivity pneumonitis which displays a strong Th17 response [11]. In contrast to BAL, there are few studies of serum cytokine profile in either disease.

Multiplexed protein analysis is increasingly used for immune profiling and biomarker identification where accurate rapid diagnosis is paramount [12] with promising data in a number of diseases including Systemic Lupus Erythematosus [13] and prostate cancer [14].

Analysis of cytokine profiles in demographically matched patients with sarcoidosis and tuberculosis may provide further insights into any relationship between both diseases or allow identification of diagnostic signatures. We therefore decided to investigate unstimulated BAL and serum samples in pulmonary sarcoidosis, pulmonary tuberculosis and healthy controls by measuring a cytokine panel which best represented the spectrum of immune process involved in both diseases including the Th1 (INFγ, TNFα) vs Th2 (IL-4, IL-5, IL-13) balance, T-cell stimulation (IL-2, IL-12), macrophage activation (Il-1b), granuloma formation (IL-8), and limitation of inflammation (IL-10). We believe that the data detailed here has achieved these aims and opens the avenue towards further work to identify serum cytokine signatures to distinguish between both diseases.

## Methods

### Ethics Statement

All participants were recruited after providing written informed consent and ethical permission for the study was obtained by the St. Mary’s Ethics Committee (reference 07/H0712/85).

### Patients and Controls

All patients with tuberculosis or sarcoidosis had pulmonary disease with evidence of additional extra-pulmonary involvement in a minority of cases. Tuberculosis samples were taken from culture confirmed cases. Sarcoidosis samples were taken from patients with a strong clinical diagnosis, supportive histology and a subsequent clinical course in keeping with the original diagnosis as per American Thoracic Society guidelines [15]. Tuberculosis and sarcoidosis samples were taken from St. Mary’s Hospital London and The Royal Brompton Hospital London, and matched to the extent possible for age, sex and ethnicity. All diseased BAL samples were taken prior to starting any treatment. Sarcoidosis serum samples were taken from patients on minimal steroid therapy (≤10 mg prednisolone daily) and on no other immunosupressants. Healthy volunteer samples were taken from two unrelated studies within the Centre for Respiratory Infection, Imperial College. All healthy volunteers were recruited from London. They were not specifically screened for latent tuberculosis disease (with either IGRA or Tuberculin Skin Testing) but they had no evidence of respiratory disease and no other significant co-morbidities. All samples were collected over a 24 month period. Demographic data for all individuals is displayed in table 1.

**Table 1:** Demographic and clinical characteristics of patients with pulmonary sarcoidosis, pulmonary tuberculosis, and healthy volunteers.

	Pulmonary sarcoidosis	Pulmonary tuberculosis	Healthy volunteers
**Bal study population**			
Number	18	12	16
Pulmonary disease only (%)	16 (89)	11 (92)	N/A
Median age (range)	47.5 (27–72)	39 (24–77)	27 (18–42)
Female gender (%)	8 (44)	6 (50)	10 (63)
Ethnic origin: Caucasian/Indian subcontinent/Black	9/2/7	6/3/3	15/0/1
Time since diagnosis	0–2 weeks	0–2 weeks	N/A
**Serum study population**			
Number	40	15	40
Pulmonary disease only (%)	29 (73)	11 (73)	N/A
Median age (range)	48 (27–73)	33 (25–71)	31 (20–60)
Female gender (%)	21 (53)	5 (33)	26 (65)
Ethnic origin: Caucasian/Indian subcontinent/Black	19/8/13	4/10/1	33/4/3
Time since diagnosis	0–5 years	0–2 weeks	N/A

### Sample Processing

Processing occurred under category 2 containment conditions. BAL fluid was collected using standard guidelines at Imperial College NHS Trust after instilling approximately 90 ml of 0.9% saline into the airways with return of approximately 40 ml. The fluid was then passed through a 100 µm aperture mesh. Serum was processed from blood which was allowed to clot for 60 mins before centrifuging at 1000 g for 10 mins at room temperature (RT). All samples were stored at −80°C within 90 mins of collection.

### Multiplex Cytokine Analysis

Multiplex cytokine analysis was performed in precoated 96 well plates (Human TH1/TH2 10 plex ultrasensitive assay, Meso Scale Discovery – MSD, Maryland, USA). 25 microL of diluent 2 was dispersed into each well. The plate was sealed and incubated by vigorous horizontal shaking for 30 minutes at RT. 25 microL of the sample (BAL fluid, serum or internal standard) was added per well and all samples measured in duplicate. Plates were sealed and incubated by vigorous horizontal shaking for two hours at RT. Plates were washed three times with 0.05% Tween 20 (Abcam, Cambridge, UK) in PBS (Sigma-Aldrich, UK). 25 microL of 1× detection antibody solution was placed per well and sealed plates were incubated by vigorous horizontal shaking for two hours at RT. Plates were washed three times with 0.05% Tween 20 in PBS. 150 microL of 2× Read Buffer T was added to each well. Plates were analysed using the MSD SECTOR Imager 2400 and Discovery Workbench 3.0 software (both from Meso Scale Discovery, USA). The mean value of two wells was taken as the recorded reading, provided that the coefficient of variation (CV) was less than 10%. Concentrations recorded lower than the standard curve were kept as absolute values. For purposes of logarithmic analysis, readings of 0 were adjusted to 0.01 pg/ml.

### Statistical Analysis

For each direct comparison (sarcoidosis vs. tuberculosis, sarcoidosis vs. healthy and tuberculosis vs. healthy) a p-value was calculated using a Mann-Whitney test for each cytokine. P-values were adjusted for multiple comparisons using a Bonferroni correction (multiplying each p-value by 10). Data analysis was performed using GraphPad Prism version 5.04 for Macintosh (La Jolla, California, USA). To create a classification rule to distinguish between diseases, partial least squares (PLS) logistic regression was carried out using the gpls package in R version 2.13.1 (R Foundation for Statistical Computing, Vienna, Austria). The within sample mis-classification rate was calculated for the logistic regression model found by PLS. Leave-one-out cross-validation was used to obtain an estimate of prediction error of the PLS logistic regression model.

**Figure 1:**
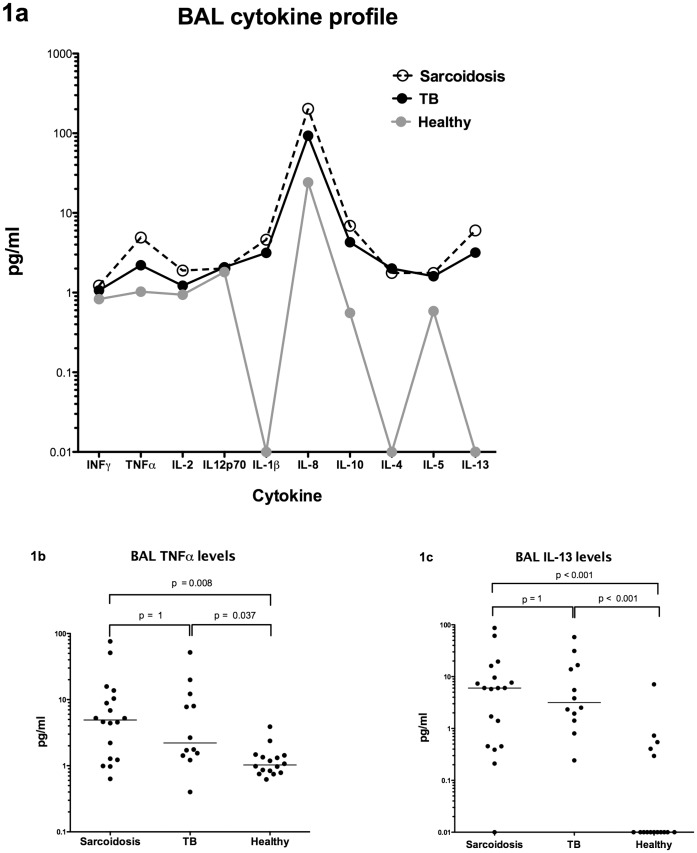
Cytokine profiles in the BAL of pulmonary sarcoidosis, pulmonary tuberculosis and health volunteers. (A) BAL cytokine profile, (B) BAL levels of TNFα and (C) BAL levels of IL-13.

## Results

### The BAL Cytokine Profile in Acute Pulmonary Sarcoidosis is Indistinguishable from Active Pulmonary Tuberculosis

The cytokine profiles in acute pulmonary sarcoidosis and active pulmonary tuberculosis BAL (all from untreated patients within 2 weeks of diagnosis) were indistinguishable from each other (Table 2 and Figure 1a). The median levels of the Th1 cytokines INFγ and TNFα were higher in both diseases compared to healthy BALs and this elevation was significant (p < 0.05) with TNFα (Figure 1b).

**Table 2:** BAL and serum cytokine levels in pulmonary sarcoidosis, pulmonary tuberculosis and healthy volunteers with Bonferroni-adjusted p-values for each of the possible comparisons.

Cytokine	Sarcoidosis BAL n = 18 median (range) pg/ml	Sarcoidosis Serum n = 40 median (range) pg/ml	Tuberculosis BAL n = 12 median (range) pg/ml	Tuberculosis Serum n = 15 median (range) pg/ml	Healthy BAL n = 16 median (range) pg/ml	Healthy Serum n = 40 median (range) pg/ml	Sarcoidosis vs. tuberculosis BAL p-value	Sarcoidosis vs. tuberculosis Serum p-value	Sarcoidosis vs. Healthy BAL p-value	Sarcoidosis vs. healthy Serum p-value	Tuberculosis vs. Healthy BAL p-value	Tuberculosis vs. healthy Serum p-value
**INFγ**	1.23 (0.21–115.5)	3.26 (0.22–42.4)	1.07 (0.42–13.03)	5.20 (0.80–74.56)	0.83 (0.01–7.01)	1.94 (0.91–37.06)	1	1	1	0.001	1	0.045
**TNFα**	4.92 (0.63–75.93)	8.73 (2.47–76.92)	2.21 (0.40–51.74)	8.92 (4.55–19.96)	1.03 (0.62–3.90)	5.31 (2.41–17.43)	1	1	0.008	<0.001	0.037	<0.001
**IL-2**	1.89 (0.20–31.12)	1.01 (0.31–3.08)	1.22 (0.48–19.67)	0.84 (0.25–1.61)	0.94 (0.42–1.8)	0.46 (0.01–10.03)	1	0.385	0.016	<0.001	0.485	0.063
**IL-12 p70**	2.01 (1.07–6.32)	3.19 (2.01–12.32)	2.08 (1.25–4.86)	2.43 (0.83–194.4)	1.81 (0.90–3.61)	2.73 (0.72–776.5)	1	1	1	1	1	1
**IL-1β**	4.62 (0.23–1167)	0.21 (0.01–11.59)	3.15 (0.45–98.48)	0.11 (0.01–1.45)	0.01 (0.01–2.84)	0.48 (0.09–180.8)	1	1	<0.001	<0.001	<0.001	0.059
**IL-8**	202.9 (1.51–5036)	10.10 (0.99–50.96)	93.20 (0.36–5155)	11.02 (3.63–22.3)	24.19 (8.07–267.8)	6.11 (1.18–2606)	1	1	0.218	<0.001	0.087	0.575
**IL-10**	6.90 (0.08–39.7)	3.48 (1.25–18.76)	4.29 (1.150–21.95)	2.33 (0.50–51.47)	0.56 (0.01–3.90)	2.86 (1.45–54.41)	1	0.838	<0.001	1	0.001	1
**IL-4**	1.77 (0.84–4.20)	0.61 (0.01–21.02)	2.01 (0.54–2.88)	0.01 (0.01–0.82)	0.01 (0.01–1.93)	1.93 (0.43–13.76)	1	0.004	<0.001	<0.001	<0.001	<0.001
**IL-5**	1.77 (0.42–58.13)	1.20 (0.58–7.92)	1.60 (0.60–49.27)	0.91 (0.01–57.91)	0.59 (0.24–2.69)	1.32 (0.39–41.81)	1	1	0.083	1	0.032	1
**IL-13**	6.03 (0.01–86.85)	0.84 (0.01–6.25)	3.17 (0.24–57.97)	0.01 (0.01–34.37)	0.01 (0.01–3.90)	1.49 (0.25–26.24)	1	0.157	<0.001	0.29	<0.001	0.014

Median BAL levels of the Th2 cytokines IL-4 and IL-13 (Figure 1c) were significantly higher in sarcoidosis and tuberculosis compared to healthy BALs (p < 0.001). Raised IL-5 levels were found in diseased BALs compared to healthy but this increase reached statistical significance in tuberculosis only. Levels of IL-1β and IL-10 were significantly elevated in both diseases compared to healthy BALs, whereas levels of IL-2 were significantly increased in sarcoidosis only. Levels of IL-12p70 and IL-8 were not significantly different between any group.

### Serum IL-4 Levels are Significantly Lower in Pulmonary Tuberculosis Compared to Pulmonary Sarcoidosis

**Figure 2:**
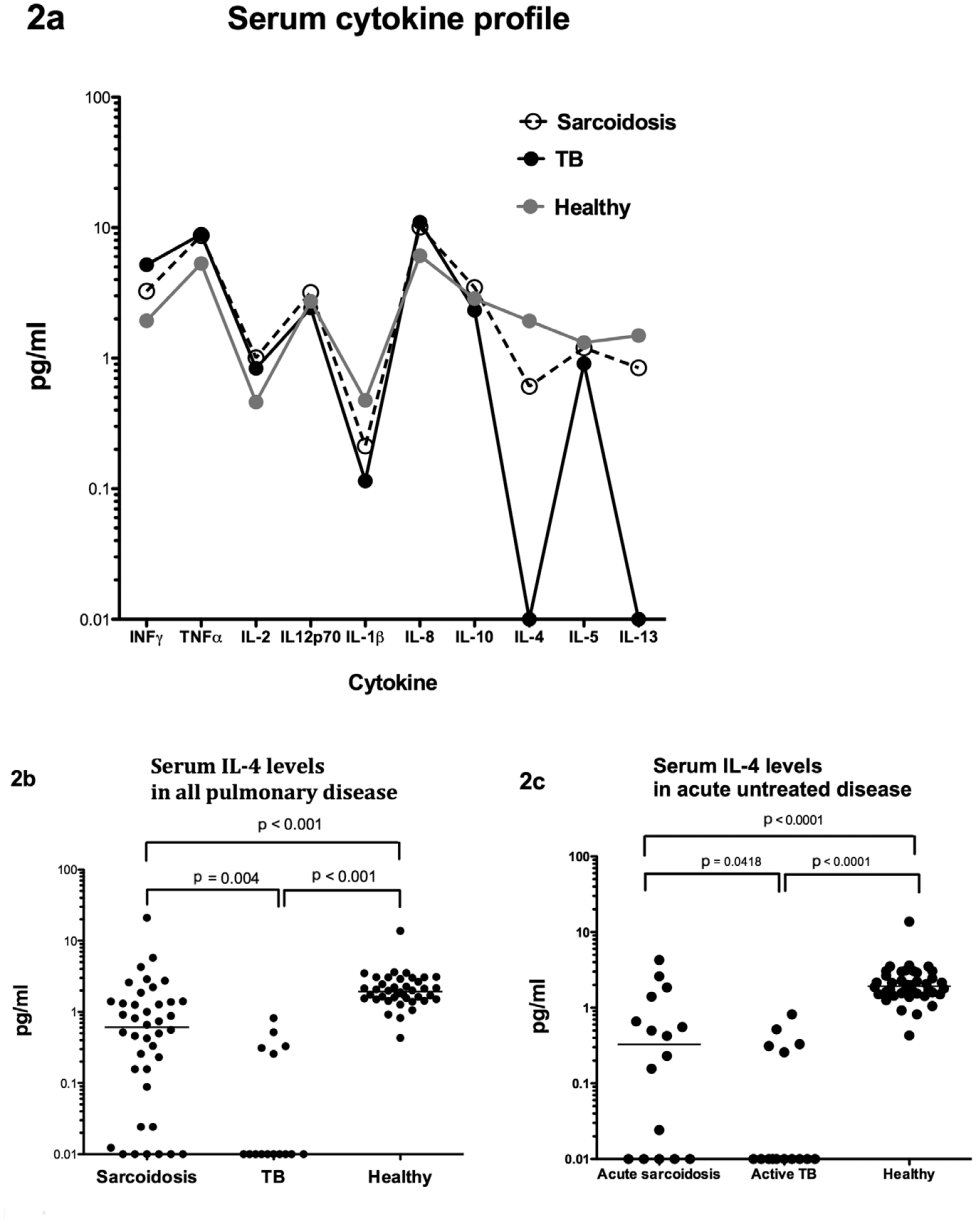
Serum cytokine profiles in sarcoidosis, tuberculosis and healthy volunteers. (A) Serum cytokine profile and (B) serum levels of IL-4 in pulmonary sarcoidosis, pulmonary tuberculosis and healthy volunteers; and (C) serum levels of IL-4 in acute pulmonary sarcoidosis, active pulmonary tuberculosis and healthy volunteers.

Serum levels of the Th1 cytokines INFγ and TNFα were significantly elevated in both pulmonary sarcoidosis and pulmonary tuberculosis compared to healthy volunteers (Table 2 and Figure 2a). Levels of IL-2 and IL-8 were elevated in both diseases compared to healthy volunteers but these increases only reached statistical significance in sarcoidosis. Lower levels of IL-1β were detected in both diseases compared to healthy serum with significance in sarcoidosis and just below significance in tuberculosis (adjusted p = 0.059). Serum levels of IL12p70 and IL-10 were not significantly different between any group.

In contrast, levels of serum Th2 cytokines IL-4, IL-5 and IL-13 were reduced in both diseases compared to the healthy volunteers. Importantly, serum levels of IL-4 were significantly lower (p = 0.004) in tuberculosis compared to sarcoidosis (Figure 2b) and these differences remained when comparing patients with acute pulmonary sarcoidosis and active pulmonary tuberculosis only, all of whom were recruited prior to the start of appropriate treatment (Figure 2c). Levels of IL-5 and IL-13 were also lower in tuberculosis although these differences did not reach statistical significance. On detailed analysis of pulmonary sarcoidosis sera, no significant differences were observed in any measured cytokine with regards to presence of pulmonary fibrosis, presence of extra-pulmonary disease or length of time since diagnosis (data not shown).

### Differences in Serum Cytokine Profile Allow for Differentiation between Pulmonary Sarcoidosis and Pulmonary Tuberculosis

A targeted bioinformatic approach to exploit the serum cytokine differences was employed to differentiate between sarcoidosis and tuberculosis. Partial least squares was used to create a logistic regression model comparing the values of all serum cytokines. The best fit model found was log(P(Tuberculosis)/P(sarcoidosis))  = 1.214 + 8.884 * IL1 + 0.075 * IFN + 0.32 * IL12p70 + 0.039 * TNF − 0.097 * IL5 − 0.015 * IL8 − 0.73 * IL13 − 0.606 * IL10 − 2.404 * IL2 − 4.231 * IL4. The classification rule was to classify a sample as tuberculosis if log(P(tuberculosis)/P(sarcoidosis)) was greater than zero, or as sarcoidosis otherwise.

**Figure 3:**
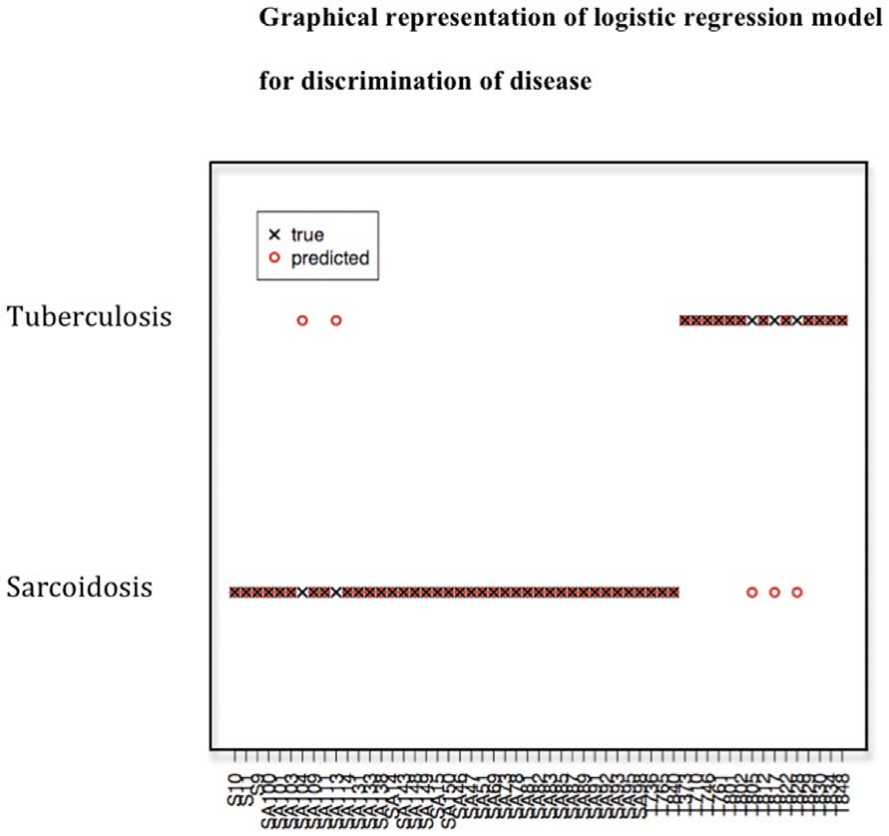
Partial Least Squares is used to create a linear regression model to accurately discriminate between sarcoidosis and tuberculosis sera.

By applying this model to the data, the observed and predicted disease groups for the sera were recorded (Figure 3). The created model had a within-sample mis-classifcation rate of 9% (5/55 sera) i.e. allowed prediction of whether a serum sample was sarcoidosis or tuberculosis with 91% accuracy. The within-sample error rate is possibly an under-estimate of the true prediction error of this model, as the same data were used both to fit the model and assess its accuracy. Leave-one-out analysis was therefore performed for cross validation and used to obtain a better estimate of prediction error which was 27% (15/55).

### Compartmentalisation of Cytokines between BAL and Serum in Sarcoidosis is Similar to that Seen in Tuberculosis

**Figure 4:**
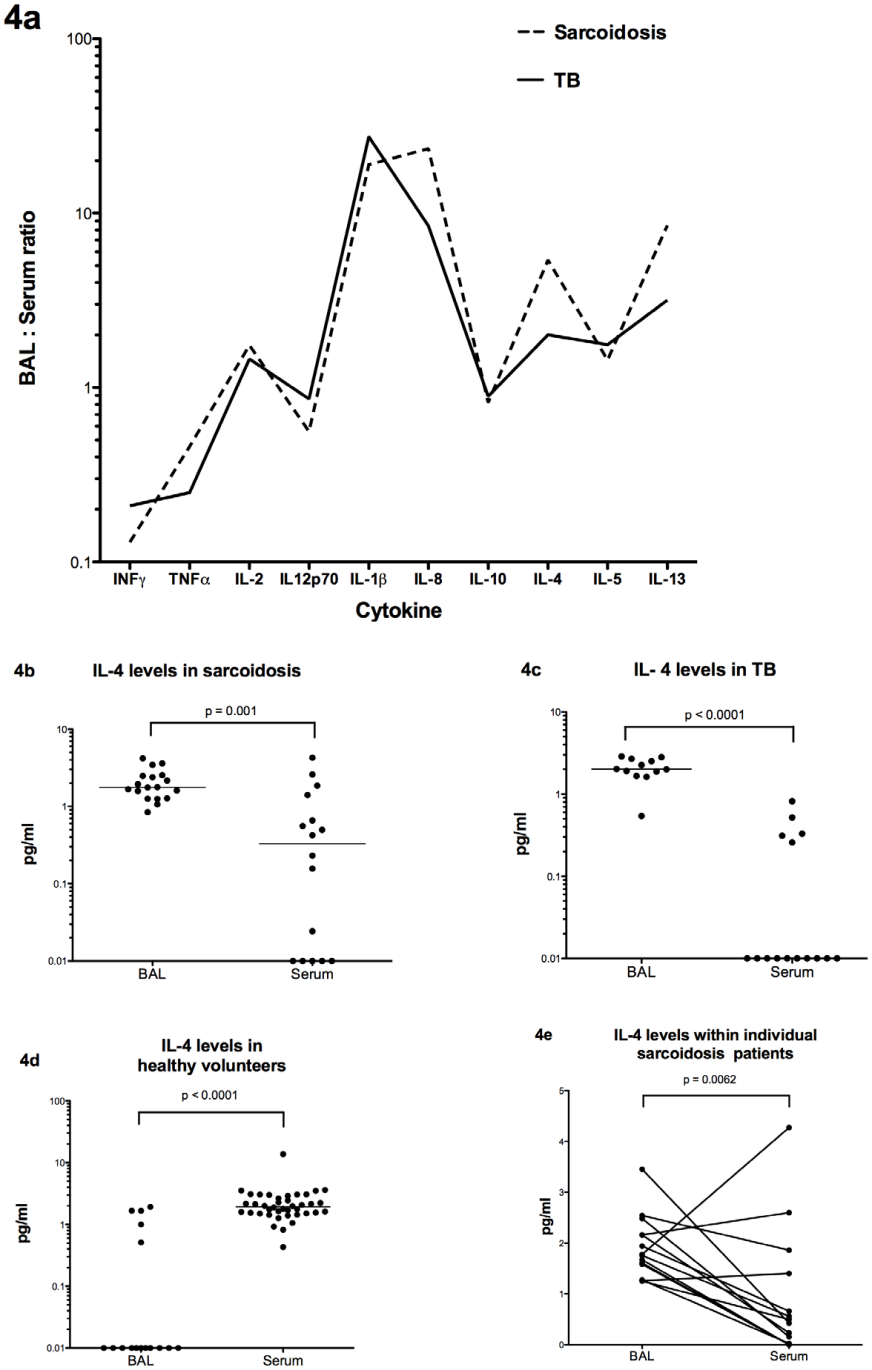
BAL: serum cytokine ratios in different conditions. (A) BAL: serum cytokine ratios seen in pulmonary sarcoidosis and pulmonary tuberculosis, (B) BAL and serum levels of IL-4 in pulmonary sarcoidosis, (C) BAL and serum levels of IL-4 in pulmonary tuberculosis, (D) BAL and serum levels of IL-4 in healthy volunteers and (E) matched BAL and serum levels of IL-4 in pulmonary sarcoidosis.

The compartmentalisation of cytokines between BAL and serum (in terms of where the higher concentration was found) was similar in acute pulmonary sarcoidosis and active pulmonary tuberculosis for all 10 cytokines measured (Figure 4a). With Th2 cytokines, differences were apparent when compared to the healthy groups e.g. median levels of IL-4 were higher in BAL than serum in both sarcoidosis (ratio of BAL: serum = 5.39) and tuberculosis (ratio = 2.01) but this pattern was reversed in healthy volunteers where almost no IL-4 was measured in the BAL giving a ratio of less than 1 (Figures 4b-d). The patterns seen were also observed where data was available within the same patients as shown in figure 4e e.g. 11 of 14 sarcoidosis patients had significantly higher levels of IL-4 in their BAL than their serum (p = 0.0062).

## Discussion

This is the first study to compare cytokine profiles both peripherally and at the site of disease in patients with sarcoidosis and tuberculosis. Acute pulmonary sarcoidosis and active pulmonary tuberculosis share a number of clinical, radiological and histological similarities making differential diagnosis difficult. We have shown that cytokine profiles in the BAL of both diseases appear indistinguishable from each other but that differences exist in serum. With respect to BAL, significantly elevated TNFα (compared to healthy volunteers) was expected from previous studies [16], [17] but is shown here for the first time to be at similar levels in demographically matched patients. This may represent a general acute phase response not seen in healthy lungs. However, TNFα has a number of specific functions including activation of macrophages and granuloma formation [18] and the indistinguishable BAL levels indicate that the host TNFα response at site of disease is similar in both conditions, regardless of the diagnosis and etiology.

The Th2 cytokines IL-4 and IL-13 were significantly raised in both diseases compared to healthy control BALs. To date, sarcoidosis BAL T-cell studies have been inconsistent with regard to Th2 cytokines. One study found levels of spontaneous IL-4 secretion similar to healthy controls [19] while another found mRNA expression of IL-13 was significantly increased compared to healthy controls [20] in keeping with our data. With regards to tuberculosis, we are unaware of previous work investigating IL-13 BAL levels but increased IL-4 secretion from both CD4+ and CD8+ T-cells in active pulmonary tuberculosis has been shown compared to those with latent infection [21]. Elevated Th2 BAL levels seen here also resulted in disease-specific compartmentalisation with levels of IL-4, IL-5 and IL-13 higher in the BAL than the serum, the opposite pattern to that seen in healthy controls.

The serum cytokine data indicate for the first time an identical Th1 cytokine pattern (as demonstrated by INFγ and TNFα) in both diseases, significantly elevated over healthy volunteers. There is limited published data on serum cytokine profiles in either disease but tuberculosis studies have found an elevated serum INFγ compared to healthy controls [22]. A major finding of our study is that serum levels of IL-4 were significantly lower in tuberculosis, compared with sarcoidosis. This difference remained after statistical bonferroni correction for multiple comparisons. The five tuberculosis patients with measurable IL-4 did not differ clinically or radiographically as a group compared to the majority with undetectable levels.

These serum differences allowed for the creation of a logistic regression model to differentiate between both diseases with 91% accuracy. Of the five patients misclassified by the model, there were no clinical features that would separate them from the correctly classified patients. Leave one out analysis was performed to cross validate our model and assess the accuracy in an independent data set and this resulted in an accuracy of 73%, perhaps representing a more accurate level of discrimination in clinical practice. A recent transcriptomic study revealed a sarcoidosis blood signature which could not be differentiated from tuberculosis [23]. Transcriptomic whole blood signatures do not necessarily correlate with serum protein levels and alternative methods of investigation, such as in the case of our study, may provide better indicators of serum cytokine activity.

One previous study has quantified mRNA levels of IL-4 in sarcoidosis and tuberculosis granulomas within diseased lymph nodes and found that four out of eight tuberculosis samples expressed detectable amounts of IL-4 and/or IL-5 mRNA, compared with undetectable levels in six sarcoidosis samples [24]. One explanation for this is that Th2 cells may localize to the site of disease in tuberculosis but not so in sarcoidosis, leading to the higher serum IL-4 levels found in our sarcoidosis patients. It is known that both diseases tend to have predominantly Th1 cells at the site of disease [8], [9] but there is currently limited published evidence for the theory that there is a difference in localization of Th2 cells between diseases. Another study showed that the intracellular IL-4 content of peripheral blood lymphocytes was diminished in patients with pulmonary tuberculosis compared to healthy volunteers [25] and a selective increase in mRNA of the IL-4 antagonist IL-4δ2 in peripheral cells has been identified in latent tuberculosis infection compared to healthy controls [26]. Both of these peripheral blood studies are consistent with our finding of very low levels of serum IL-4 in the majority of patients with pulmonary tuberculosis.

A possible explanation for the similarities in BAL but differences in serum cytokine profiles is that both sarcoidosis and tuberculosis share similar infectious triggers leading to the identical granulomatous-type host immune responses in the lungs. This is supported by indistinguishable levels of all cytokines measured in the BAL spanning the spectrum of pathogen response including T-cell stimulation (IL-2, IL-12), macrophage activation (IL-1b) and granuloma formation (TNFα, IL-8). However, despite similar local responses at the site of disease, the systemic response varies between individuals as evidenced by significantly higher serum IL-4 levels (and the possible elevation of IL-5 and IL-13) in sarcoidosis. The increased serum IL-4 levels in sarcoidosis may reflect a persistent and ongoing multi-system inflammatory allergic type response to an antigen not completely cleared by the immune system, in contrast to the lower levels seen in tuberculosis. Importantly, there was no difference in incidence of asthma or COPD between the sarcoidosis and tuberculosis groups so this is not responsible for the differences seen in serum Th2 profiles.

A number of diseases of abnormal host immunity such as chronic graft vs host disease [27] and systemic sclerosis [28] are characterised by Th2 cells secreting IL-4 which subsequently plays a role in the activation and recruitment of B cells producing IgE, mast cells and eospinophils. The higher IL-4 levels observed in sarcoidosis may therefore also account for the peripheral antibody response which is stronger than that seen in tuberculosis [29]. Elevated serum IL-4 in sarcoidosis suggests that the use of selective IL-4 antagonists or soluble recombinant IL-4 receptors could be investigated for their efficacy to reduce disease activity in sarcoidosis [30]. Detailed investigations into peripheral IL-4 levels in sarcoidosis may also further our understanding into the mechanisms of action of immunosupressent therapy in the disease.

Our study has several limitations. Firstly, given the inherent intragroup variability of BAL cytokine levels seen in disease states, a much larger analysis will be needed to exclude the possibility of further differences being found between sarcoidosis and BAL cytokine profiles. This is particularly important for those cytokines where the median level is low, despite there being a large detected range e.g. when measuring levels of INFγ and IL-8 in the BAL of sarcoidosis and tuberculosis patients. A larger data set will also be able to determine the immunological or clinical relevance of any outliers at the higher range of the cytokine measurement. As part of a larger study, it would be interesting to include patients with other granulomatous diseases such as Wegener’s granulomatosis. This could determine whether there is a cytokine profile that reflects granulomatous lung conditions in general. Secondly, the stage of disease (as determined by pulmonary radiograph) in patients with sarcoidosis was not determined for this study as our experience has shown us that even expert radiologists can make subjectively differing assessments of stage when assessing the same radiographs. However, presence or absence of pulmonary fibrosis through High Resolution CT scanning of the chest was not associated with BAL or serum cytokine profile in sarcoidosis (data not shown). Finally, all BAL samples were taken from patients in the acute stage of their disease prior to commencing appropriate therapy but sera were taken from patients with a longer disease duration, some of whom were on treatment. Despite minimal maintenance doses of immunosuppression, we cannot exclude the possibility that even low doses may have affected the cytokine profile.

However, a comparison of patients with acute untreated pulmonary sarcoidosis (n = 16) and those with more chronic disease (n = 24), some of whom may have been on steroid therapy, shows that there were no differences in any serum cytokine measured, indicating no significant effect of immunosuppression on the serum cytokine profile in sarcoidosis (data not shown). Furthermore, the main difference we identified between sarocidosis and tuberculosis, ie serum IL-4 levels, remains statistically significant after correction for multiple comparisons even when only patients with acute untreated pulmonary sarcoidosis and active pulmonary tuberculosis were compared (figure 2c). All these samples were taken from patients at the time of bronchoscopy, prior to starting treatment for either tuberculosis or sarcoidosis. The significant difference in serum IL-4 levels between the two diseases is therefore not an artifact of treatment.

There are currently no consistently reliable blood tests to either diagnose sarcoidosis or to distinguish between sarcoidosis and tuberculosis. Serum Angiotensin Converting Enzyme (ACE) levels measured in sarcoidosis to monitor disease progression (which are the only serum biomarker in routine clinical use) are non-specific as ACE is elevated in a number of diseases including tuberculosis itself [31]. After analysis of a broad spectrum of immune biomarkers (including Th1, Th2 and granuloma-associated cytokines) in both BAL and serum, we have shown for the first time that only one cytokine in one compartment, serum IL-4, is significantly different between sarcoidosis and tuberculosis and that this difference holds up under strict statistical correction for multiple comparisons. We have also shown that this difference is still present when analysing patients at first presentation with acute pulmonary disease, prior to any treatment, as would be found in the clinical setting where rapid and accurate diagnosis is paramount.

Despite these findings, the single measurement of serum IL-4 will not suffice for differential diagnosis between diseases. Despite the differences being statistically significant, there is still considerable overlap between the levels measured in individual patients with sarcoidosis and tuberculosis. However, the differences seen with IL-4 may form the basis of a broader serum cytokine profile to accurately distinguish sarcoidosis from tuberculosis; incorporating additional markers in a larger study might substantially improve on the 73% accuracy of the panel used here. Such future studies should focus only on newly diagnosed tuberculosis and sarcoidosis patients in the acute stage of disease, prior to starting any therapy, as these groups are where disease differentiation is paramount. Such an approach may prove a rapid adjunct to differential diagnosis which could be employed in the clinical setting prior to any invasive procedures. Furthermore, systemic Th2 cytokine differences between the two diseases may underpin different disease outcomes in patients exposed to similar respiratory stimuli.
